# On the Use of Full Doses of Opium in Continued Fevers

**Published:** 1852-07

**Authors:** 


*On the Use of Full Doses of Opium in Continued Fevers.* By Dr. Haycraft.—(London Lancet.) Dr. Haycraft considers that the administrntion of opium, in the latter stages of continued fever, is a practice which has fallen into undeserved disuse, which, he remarks, is the more unaccountable, inasmuch as the usual effect of fever is to deprive the patient of sleep. He argues that, if any one were to be deprived of sleep for ten, twelve or fifteen days, he would be, by that time, in a state somewhat similar to that of the exhaustion of fever.

"By what train of reasoning (he asks) can we come to the conclusion that sleep is less necessary in disease than in health? That the occurrence of sleep is highly salutary in fever requires for proof (Dr. Haycraft adds) only the concurrent testimony of all observers, provided that the sleep be not of the comatose kind. Even the nurses of our public institutions will frequently form a favorable and correct prognosis, when they say, in hospital phrase, "the patient is sleeping an end," and the event seldom falsifies the prediction."

Our author examines into and comments on the opinions of Sydenham, Van Swieten, Boerhaave, Friend, Richter, Cullen, Mason Good, Potter, Armstrong, etc., as to the employment of opium in fever. He remarks that both Sydenham and Van Swieten mention delirium as the symptom which indicates its use; but that, from most of the authors above named "placing this drug at the *end* of their lists, it might fairly be implied that it is only to be given at the termination of the disease—certainly not at its commencement."

The presence of inflammation is far from contra-indicating the use of opium in fever. Drs. Stokes and Graves have shown that, in peritonitis, the inflammation may be subdued by the use of opium alone, even when the intestine has been perforated.

Some of the conclusions of Dr. Stokes, as quoted by Dr. Haycraft, are, that where antiphlogistic measures are inadmissible, the administration of opium in large doses may be made with benefit, and that its effect is then to raise the vital power. Our author believes that this effect may be "partly due to the production of sleep, which, however induced, must act beneficially on depressed vitality; that it should subdue irritability may, I think, (says Dr. Haycraft,) be fairly attributed to the same operation."

Dr. Haycraft lays great stress on the injunction, that the remedy should be administered in *full* doses. A small dose, as he avers, will only irritate, and therefore increase restlessness and delirium; and the later opium is used, in the progress of the fever, the more decided, he considers, will be its beneficial effects; if given on the fourteenth day from the attack, it will, he says, usually produce a most decided critical sleep: "the fever is actually cut short." Earlier than the eighth day, he states that he has, from the nature of his practice, not had opportunities of giving it; but he would not hesitate to employ it after the sixth day, provided *restlessness*, *sleeplessness*, or *delirium* were present. This treatment of fever by opium is not, however, he cautions us, to supersede other remedies, which may be specially required to combat concurrent symptoms—wine, brandy, diffusible stimulents when exhaustion takes place, sulphuric acid in large diluted doses, when colliquative sweats exist, or a scruple of calomel, with or without opium, when the congestion of collapse occurs in the brain, lungs or abdomen. He does not consider opium, in suitable doses, a remedy in anywise contra-indicated for children suffering from fever.

Dr. Haycraft cites several cases, which have occurred in his practice, illustrative of his views. The following are the conclusions at which he has arrived:

"1st. That opium may be safely given after the sixth or eighth day of a continued fever.

"2d. That it will be most beneficially prescribed when restlessness, insomnolentia, delirium or sinking takes place.

"3d. That a drachm of the tincture of opium, or an equivalent quantity of any other preparation of opium, is not too large a quantity for an adult.

"4th. That opium, being an antiphlogistic remedy, is not contra-indicated by inflammation.

"5th. That opium succeeds best after purgatives—that if these have been neglected it should be joined with a purgative; if the symptoms urgently require its use, it may be combined with diuretics.

"6th. That opium, given in the latter stages of fever, in sufficient doses, raises the vital powers, abates irrita-

